# Protein disorder prediction by condensed PSSM considering propensity for order or disorder

**DOI:** 10.1186/1471-2105-7-319

**Published:** 2006-06-23

**Authors:** Chung-Tsai Su, Chien-Yu Chen, Yu-Yen Ou

**Affiliations:** 1 Department of Computer Science and Information Engineering, National Taiwan University, Taipei, 106, Taiwan, R.O.C; 2 Department of Bio-industrial Mechatronics Engineering, National Taiwan University, Taipei, 106, Taiwan, R.O.C; 3 Graduate School of Biotechnology and Bioinformatics, Yuan Ze University, Chung-Li, 320, Taiwan, R.O.C; 4 Department of Computer Science and Engineering, Yuan Ze University, Chung-Li, 320, Taiwan, R.O.C

## Abstract

**Background:**

More and more disordered regions have been discovered in protein sequences, and many of them are found to be functionally significant. Previous studies reveal that disordered regions of a protein can be predicted by its primary structure, the amino acid sequence. One observation that has been widely accepted is that ordered regions usually have compositional bias toward hydrophobic amino acids, and disordered regions are toward charged amino acids. Recent studies further show that employing evolutionary information such as position specific scoring matrices (PSSMs) improves the prediction accuracy of protein disorder. As more and more machine learning techniques have been introduced to protein disorder detection, extracting more useful features with biological insights attracts more attention.

**Results:**

This paper first studies the effect of a condensed position specific scoring matrix with respect to physicochemical properties (PSSMP) on the prediction accuracy, where the PSSMP is derived by merging several amino acid columns of a PSSM belonging to a certain property into a single column. Next, we decompose each conventional physicochemical property of amino acids into two disjoint groups which have a propensity for order and disorder respectively, and show by experiments that some of the new properties perform better than their parent properties in predicting protein disorder. In order to get an effective and compact feature set on this problem, we propose a hybrid feature selection method that inherits the efficiency of uni-variant analysis and the effectiveness of the stepwise feature selection that explores combinations of multiple features. The experimental results show that the selected feature set improves the performance of a classifier built with Radial Basis Function Networks (RBFN) in comparison with the feature set constructed with PSSMs or PSSMPs that adopt simply the conventional physicochemical properties.

**Conclusion:**

Distinguishing disordered regions from ordered regions in protein sequences facilitates the exploration of protein structures and functions. Results based on independent testing data reveal that the proposed predicting model DisPSSMP performs the best among several of the existing packages doing similar tasks, without either under-predicting or over-predicting the disordered regions. Furthermore, the selected properties are demonstrated to be useful in finding discriminating patterns for order/disorder classification.

## Background

A large proportion of proteins are found to be intrinsically disordered, which refers to segments of protein sequences that do not form well-defined three-dimensional structures in their native states [1-6]. Many proteins contain local regions of such disorder, and in some cases entire proteins appear to exist as ensembles of structures [1-3,7,8]. Since protein subsequences that lack the ability to form a well-defined three-dimensional structure may still be functionally related, identifying such disordered regions is getting more and more important for both understanding protein functions and conducting structural analyses [6].

Protein disorders have been observed in a variety of biological functions, including molecular recognition, cell signaling pathways, and enzyme catalysis [3,9]. Taking the yeast proteome as an example, the proteins containing disorder are often located in the cell nucleus and are involved in transcription regulation and cell signalling [4]. The disordered regions that remain as flexible ensembles under physiological conditions allow the disordered binding sites to interact with several different targets [10,11]. The disorder-to-order transition upon binding provides high specificity with modest binding affinities [7,12].

It has been shown in many studies that protein disorders can be predicted from their primary sequences [10,13-18]. The prediction methods developed in recent years initiate the possibility of identifying such disordered binding sites automatically [14,17]. A more general concept is that all the necessary information for the correct folding of a protein is included in its amino acid sequence [19]. Disordered regions are comprised of a category of amino acids distinct from that of ordered protein structures [20]. For example, amino acids of aromatic hydrophobic groups are known to be good for the general stabilization of order, and thus are less found in the disordered regions [13]. Incorporating information of the biased amino acid composition in a neural network predicts the locations of disorder with accuracy better than random guesses [13]. In 1998, Romero *et al*. showed that more than 15,000 proteins in the Swiss-Prot database contain long disordered segments (40 or more residues) based on their predictions [14,21]. Studies on some of these disordered regions reveal that they are evolutionarily conserved and possess biological functions [10].

Several machine learning approaches, such as neural networks (NNs) [6,10,13,22], logistic regression (LR) [22,23], discriminant analysis (DA) [23], ordinary least squares (OLS) [22], and support vector machines (SVM) [4,23,24] have been introduced to protein disorder prediction. Since different classifiers deliver similar prediction accuracy based on the same feature set [23], extracting more useful features with biological insights to improve the quality of prediction attracts more attention in recent studies [10,24]. As amino acid composition has been demonstrated as a useful feature for detecting disordered regions, Jones *et al*. showed in their paper that using the position specific scoring matrices (PSSMs) within a specific length of window centred at a given residue can improve the accuracy of predicting its disorder attribute [10]. The values in a position specific scoring matrix indicate the level of conservation of a position and the properties of the substituted residues, which can be derived directly from executing PSI-BLAST for each target protein sequence. PSSMs have been demonstrated to be powerful in constructing feature sets for prediction of single-residue properties from an amino acid sequence, such as category of secondary structures or solvent accessibility [10]. The evolutionary information summarized in a PSSM table generalizes the attribute of each position in a protein sequence, and thus improves the sensitivity of the predicting model.

A development of this approach employs a condensed position specific scoring matrix with respect to physicochemical properties (it will be called PSSMP in this paper) in predicting protein disorder, where the PSSMP is derived by merging several amino acid columns of a PSSM that belong to a certain property into a single column [24]. As a PSSM brings in the evolutionary information on each position, a PSSMP summarizes this information as property attributes. The improvement achieved by PSSMP demonstrates that property attributes are more informative than amino acid attributes in distinguishing ordered/disordered regions. A more comprehensive study conducted in this paper reveals that PSSMPs outperform PSSMs especially when the employed window size is large.

When employing PSSMP tables as the feature set in protein disorder prediction, two questions arise: (1) if all the amino acids in one physicochemical property group contribute to the predicting power; and (2) if all the amino acids in one physicochemical property group result in consistent effect on prediction. It has been widely studied in previous works that the propensity for order or disorder of several amino acids is clear. Hydrophobic amino acids are more frequently observed in ordered regions than disordered regions [18,19]. Among them the aromatic amino acids are present in different locations to the aliphatic amino acids [25]. On the other hand, the amino acids with charge imbalance are often present in disordered regions. In this paper, we argue that the propensity for order or disorder of each amino acid should be considered when constructing PSSMP. After examining the statistics derived by comparing the sequence segments in ordered regions and disordered regions, we observe that not all the hydrophobic amino acids possess a propensity for order. Thus we suggest that each conventional physicochemical property should be divided into two smaller groups with propensities for order and disorder respectively, such as hydrophobic with an order propensity (*Hydrophobic*_*O*_) and hydrophobic with a disorder propensity (*Hydrophobic*_*D*_). The experiments conducted in this work reveal that some newly derived properties provide more accurate information regarding order or disorder.

Incorporating the propensity for order/disorder with physicochemical properties in PSSMP produces informative features for protein disorder prediction. However, the number of candidate features becomes larger than in the case when only twenty amino acids are considered. The size of the feature set gets even larger when a large window size is employed, which might cause the performance of the learning algorithms to be degraded due to abundant noise. Thus, we present in this paper a feature selection mechanism that considers both the size and effectiveness issues when determining a feature set on protein disorder prediction. A wrapper approach of feature selection is employed during training period that invokes the adopted Radial Basis Function Networks (RBFN) classifier to evaluate the predicting power of a candidate feature set. A cluster-based redundancy removal procedure is incorporated to speed up the stepwise feature selection process, where two levels of redundancy among features are considered.

As far as the experimental materials are concerned, a new dataset PDB693 organized from the Protein Data Bank (PDB) [26] database is coined in this work to benefit the study on protein disorder. PDB693 and another dataset D184 collected from Database of Protein Disorder (DisProt) [27] constitute the training data of our classifier DisPSSMP. The performance of DisPSSMP is compared with twelve existing disorder prediction packages, where the blind testing data comes from a recent study [6]. The experimental results demonstrate that the selected property features are informative in protein disorder prediction and can be used to derive discriminating patterns for order and disorder classification.

## Results and discussion

In this section, we first describe how the datasets have been prepared and how the performance of prediction is evaluated. Next, we show the results of the feature selection after conducting cross-validation on the training data. At this stage, we also discuss the effect of the window size employed in constructing PSSMP. After that, the resultant feature set is employed in constructing the final predicting model DisPSSMP. Finally, the testing results are evaluated based on the bind testing data, and are compared with other existing packages performing similar tasks. At the end of the section, we show the derived property sets can be used to discover patterns that distinguish ordered and disordered regions.

### Datasets

In this study, five datasets have been collected or newly created for training and validating processes. The detailed statistics about each dataset are provided in Table 1, including the number of chains, ordered/disordered regions, and residues in ordered/disordered regions. The training data used in constructing the predictor DisPSSMP is composed of datasets PDB693 and D184, which are respectively organized from PDB and DisProt database based on the procedures described in the following paragraphs. Meanwhile, three datasets named R80, U79, and P80, which are taken from two related studies [6,19], constitute an independent testing data. This blind dataset serves as a platform for comparing the performance of the proposed method with some other existing packages performing protein disorder prediction.

The dataset PDB693 contains 693 partially disordered proteins, where the locations of disordered regions are identified by looking for the missing residues in a protein structure from PDB database (28-Aug-2005 version). There are originally 32204 structures in this version of PDB database, and those structures are filtered by a clustering program Cd-Hit [28,29] such that the resultant nonredundant set containing no pair of protein sequences with similarity identity of more than 70%. The so-called missing residues are those present in the SEQRES records but not in the ATOM records with their alpha-carbon coordinates. A protein sequence is considered in this study only if it contains at least one disordered region with more than 30 consecutive residues. Furthermore, protein sequences of similarity identity of more than 70% against any protein sequence in the independent testing data have been removed, resulting in 693 protein sequences in the PDB693 dataset.

Another training set D184 is extracted from DisProt database. DisProt is a curated database that provides information about proteins that entirely or partially lack a fixed three-dimensional structure under putatively native conditions [27]. The DisProt release 2.2 consists of 202 proteins, including 431 distinct disordered regions in total. Among the 202 proteins, there are 157 proteins that contain at least one disordered region longer than 30 consecutive residues. There are more than 50 wholly disordered proteins in DisProt database which are annotated as serving certain functions. D184 is also filtered by Cd-Hit to remove redundant proteins which have more than 70% identity with some other proteins inside it or in any of the three testing datasets.

The dataset R80 was prepared by Yang *et al*. in 2005 [6]. The 80 protein chains in this dataset are collected from the PDB database, and each protein chain contains a region of at least 21 consecutive disordered residues. Additionally, the dataset U79 organized by Uversky *et al*. in 2000 [19] and the dataset P80 provided by PONDR^® ^web site (retrieved in February 2003) are also compiled into the blind testing set, where the dataset U79 contains 79 totally disordered proteins and the dataset P80 contains 80 completely ordered proteins. By using Cd-Hit again, we observed that two sequences in P80 are subsequences of a protein in R80 and a pair of proteins in U79 have identity of 73%. Like Yang *et al*. did in their paper [6], these three datasets are employed as a platform for comparison of our approach to some other present packages targeting at protein disorder prediction. Thus, we did not change the contents of these three datasets such that the comparison can be carried out. In particular, the datasets U79 (fully disordered proteins) and P80 (globular proteins) together suggest whether the proposed method is under- or over-predicting protein disorder.

### Evaluation measures

Predicting a residue in the given protein sequence as order or disorder is a binary classification problem, and many measures have been introduced for validation issues [30,31]. Table 2 lists four widely used indices defined by previous related works [6,18,24,30-32]. We employ these measures in this study to evaluate the performance of different feature sets or different packages. *Sensitivity *represents the fraction of disordered residues correctly identified in a prediction method, while *specificity *indicates the fraction of ordered residues correctly identified. The *Matthews' correlation coefficient *is a popular measure in many bioinformatics problems [33-35]. However, *sensitivity*, *specificity*, and the *Matthews' correlation coefficient *are seriously affected by the relative frequency of the target class. Therefore, the above three measures are not suitable for evaluating the performance in isolation. The *probability excess *is independent of the relative class frequency, and this measure can be reduced to *sensitivity *+ *specificity *- 1 concisely [6]. In addition, some other indices including the *CASP S score*, *product*, and *probability excess *are recommended and advised by CASP6 [31] and Yang *et al*. [6] for evaluating the performance of prediction. Since these three measures have the same tendency with *probability excess*, we adopt only the *probability excess *in this paper for simplicity and show the results of other measures in the supplementary [See Additional file 1].

### Feature selection by cross-validation

In order to conduct a five-fold cross validation, the chains in datasets PDB693 and D184 are randomly split into five subsets of approximately equal size. The results of uni-variant analysis on each property feature are shown in Table 3, in which the properties oriented from the same physicochemical group are put together for the following dependency analysis. The dependency analysis of feature selection aims to answer if a subset of a property group performs better than the original one.

It is observed in Table 3 that the performance of some physicochemical properties has been improved after they are split into order/disorder-based properties. In other words, purifying the physicochemical properties by considering the propensity for order or disorder contributes to the predicting power of the classifier. *Hydrophobic*_*O *_is the best property among all of them and gets an explicit improvement over *Hydrophobic*. On the other hand, neither *Polar*_*D *_nor *Polar*_*O *_get a better performance than *Polar*. In summary, the decomposition of some conventional properties by considering the order/disorder propensity brings explicit benefit in terms of the uni-variant analysis.

**Figure 1:**
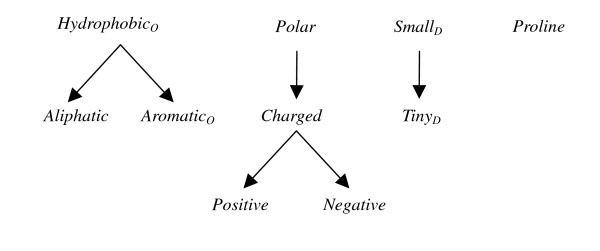
The relation of the selected properties after the first level of redundancy analysis.

After the best property for each group has been determined, a second level of dependency analysis is performed by considering the relations between physicochemical properties. The selected features are shown in Figure 1, and the relation of these features is derived by incorporating the inheritance relationships between the child properties and their parent properties. That is, *Aliphatic *and *Aromatic*_*O *_are subsets of *Hydrophobic*_*O*_, *Tiny *is a subset of *Small*, *Positive *and *Negative *are subsets of *Charged*, which recursively is a subset of *Polar*. Based on these hierarchies, we aim to investigate if a combination of two subproperties performs better than the original one. According to the results shown in Table 4 and Table 5, property features *Aliphatic*+*Aromatic*_*O *_performs better than *Hydrophobic*_*O*_, but *Positive*+*Negative *is not superior to *Polar*.

After the dependency analysis, the redundancy removal step selects the best property from each cluster for the next step of feature selection. The selected representative properties are sorted by their performance in the uni-variant analysis, resulting in the following order: *Aliphatic+Aromatic*_*O*_, *Polar*, *Small*_*D*_, and *Proline*. The stepwise feature selection is preformed by adding one candidate property in each iteration until the predicting performance cannot be improved. The results of the stepwise feature selection are shown in Table 6, indicating that the four properties, *Aliphatic*, *Aromatic*_*O*_, *Polar*, and *Small*_*D*_, will be used in constructing the final RBFN classifier for predicting protein disorder. We name the final feature set of PSSMP with four properties as FS-PSSMP-4, the feature set of ten conventional physicochemical properties as FS-PSSMP-10, and the feature set employing the original PSSM as FS-PSSM.

### Suggestion of window size

All the experiments described above have been conducted with different window sizes of 11, 35, and 59, and the resulted feature sets are the same as reported. However, though they turn out the same result on feature selection, it is observed that larger window sizes such as 35 and 59 are favourable when prediction accuracy is considered. The current version of DisPSSMP adopts a window size of 47 and thus employs in total 4 × 47 = 188 attributes in the feature vector for a query residue.

### Results on testing data

**Figure 2:**
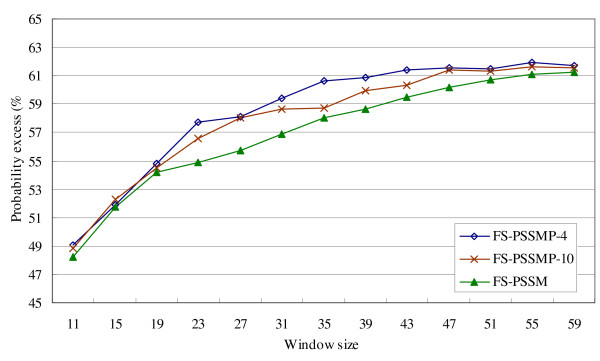
Comparison of using different feature sets on testing data R80.

**Figure 3:**
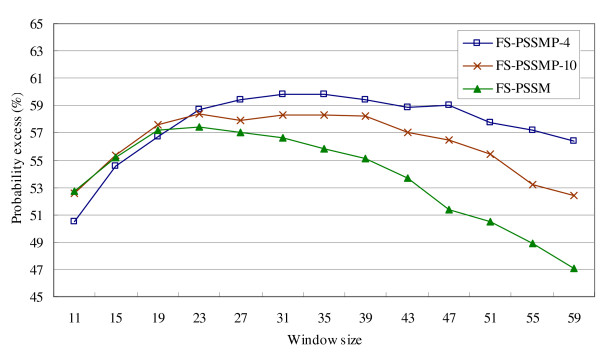
Comparison of using different feature sets on testing data U79 and P80.

In this subsection, we compare the performance of three feature sets, FS-PSSM, FS-PSSMP-10, and the proposed FS-PSSMP-4 on the independent testing data, which consists of three datasets, R80, U79, and P80. In the following discussions, the results on datasets U79 and P80 are always combined when they are reported, because U79 contains only fully disordered proteins and P80 comprises only completely ordered proteins. The results are shown in Figure 2 and Figure 3, with the window size changes from 11 to 59 systematically. It is clearly shown in Figure 2 that larger window sizes deliver better performance for all the feature sets on the dataset R80, and the performance of FS-PSSMP-4 and FS-PSSMP-10 are generally better than FS-PSSM.

**Figure 4:**
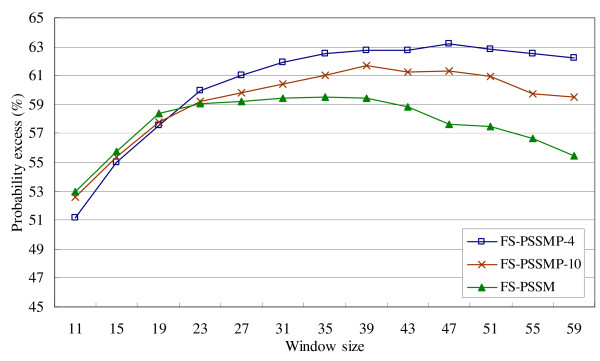
Comparison of using different feature sets on testing data R80, U79, and P80.

On the other hand, the *probability excesses *of all the feature sets in Figure 3 decrease considerably when the window size is more than 39 due to the over-prediction in terminal regions. In fact, FS-PSSMP-4 performs worse than FS-PSSMP-10 and FS-PSSM when the window size is smaller than 19, but better than both of them when the window size is larger than 23. Figure 4 shows the overall performance by combining these three testing datasets. The average performance of FS-PSSMP-4 delivers the highest *probability excess *among the three feature sets. Also, FS-PSSMP-4 has the fewest number of features among all. To sum up, FS-PSSMP-4 is superior to the others for its success of feature reduction and the improvement of accuracy and efficiency, and it delivers roughly the same level of accuracy when the window size is larger than 35.

### Comparison with existing packages

**Figure 5:**
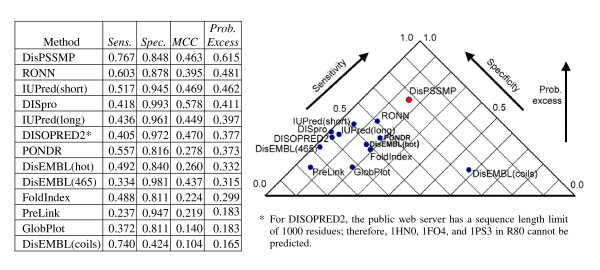
Comparing the performance of thirteen disorder prediction packages on testing data R80.

**Figure 6:**
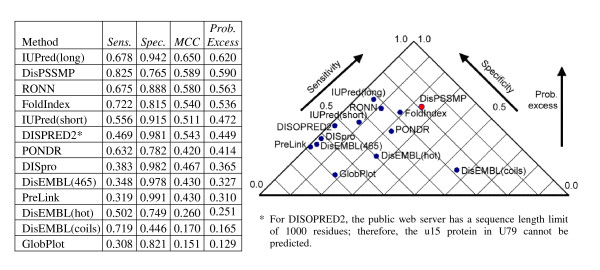
Comparing the performance of thirteen disorder prediction packages on testing data U79 and P80.

**Figure 7:**
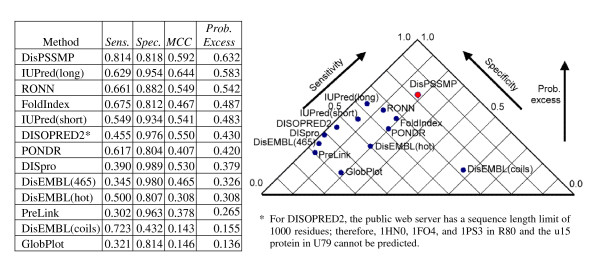
Comparing the performance of thirteen disorder prediction packages on testing data R80, U79, and P80.

In this subsection we investigate the performance of twelve web servers or packages in protein disorder prediction, some of which were included in comparison with the work of Yang *et al*. in their paper [6]. The predictors for comparison here are RONN [6], IUPred(short) [36,37], IUPred(long) [36,37], DISpro [38], DISOPRED2 [4,32], PONDR^® ^[21], DisEMBL(hot) [39], DisEMBL(465) [39], FoldIndex [19,40], PreLink [18], GlobPlot [41], and DisEMBL(coils) [39]. DISOPRED2 has a limit of 1000 residues per protein, so 1HN0, 1FO4, and 1PS3 in dataset R80 and the u15 protein in dataset U79 have been removed from the blind testing data when testing DISOPRED2. IUPred provides two choices of predicting short or long disordered regions, and DisEMBL provides three choices: DisEMBL(hot), DisEMBL(465), and DisEMBL(coils). The plots in Figure 5, Figure 6, and Figure 7 show the results in the way of *specificity *versus *sensitivity*, and the plots are rotated anticlockwise by 45° to be equivalent to the plot of *probability excess *= *sensitivity *+ *specificity *- 1.

When compared with the other packages, DisPSSMP performs the best when *probability excess *is considered (with a *probability excess *of 0.60). In Figure 5, DisPSSMP shows its ability in identifying the boundaries of ordered and disordered regions. The predictors IUPred(long), DISpro, DISOPRED2, DisEMBL(465), and PreLink have a *specificity *of more than 95% but a *sensitivity *of less than 50%, which show the tendency of predicting order more than disorder. In contrast, the predictor DisEMBL(coils) with a *sensitivity *of less than 50% but a *specificity *of more than 70% has the tendency of predicting disorder more than order. It depends on the users to select the predictors IUPred(long), DISpro, DISOPRED2, DisEMBL(465), and PreLink for under-prediction of disorder and DisEMBL(coils) for over-prediction.

The main purpose of the experiment on datasets U79 and P80 is checking whether a method is under-predicting or over-predicting protein disorder. As shown in Figure 6, the results of all the methods except IUPred(long) and FoldIndex are similar to that in the main blind testing dataset R80 in Figure 5. The *sensitivity *of IUPred(long) and FoldIndex have an improvement of more then 20% in this experiment, and they are ranked as the first and the fourth among all methods. Since IUPred(long) has been designed for predicting context-independent global disorder that encompasses at least 30 consecutive residues in the predicted disordered regions and adopts a large window size like 101 [36,37], it is suitable for the recognition of the fully globular proteins and the totally unstructured proteins. On the other hand, the training data of FoldIndex contains 91 totally unfolded proteins and 275 globular proteins, resulting in its good performance in discriminating fully ordered proteins from fully disordered proteins [19,40]. Nevertheless, due to the lack of information about the boundaries between ordered regions and disordered regions, FoldIndex does not have a good performance in R80.

The overall comparison is provided in Figure 7 by combining the results of Figure 5 and Figure 6. There are only three methods that have a *probability excess *of more than 0.50. Although IUPred(long) has an distinguishing performance in the combined blind test, this method cannot predict short disordered regions correctly due to the constraint of its algorithm. DisPSSMP and RONN have comparable performances in identifying disordered residues or ordered residues when no prior information is available.

### Property-based sequential patterns

In the previous subsections we have demonstrated that the newly defined property groups perform better than the original physicochemical properties when incorporated with position specific scoring matrices in predicting protein disorder. In this subsection, we show the potential of the newly defined property features from another point of view. As most approaches of protein disorder prediction use the subsequences centered at the target residue as discriminative features, it is generally believed that sequential patterns of amino acids may provide useful information about protein order and disorder [4,10,17,23,39,42]. Lise *et al*. also found some reliable and significant sequence patterns that characterize disordered segments [5]. Here, we show by examples of sequence patterns that the selected feature properties indeed serve as better units in characterizing both ordered and disordered regions.

In this experiment, the ordered and disordered regions are extracted from both the training and testing datasets and are organized as two datasets respectively. The scoring function employed by Lise *et al*. [5] is adopted here for evaluating the discriminating power of patterns. The score *S*_*o *_or an alternative score *S*_*d *_are defined as in Eq. (1) to represent the ordered and disordered preference of a pattern.

So=MoNoMoNo+MdNd and Sd=1−So,     (1)
 MathType@MTEF@5@5@+=feaafiart1ev1aaatCvAUfKttLearuWrP9MDH5MBPbIqV92AaeXatLxBI9gBaebbnrfifHhDYfgasaacH8akY=wiFfYdH8Gipec8Eeeu0xXdbba9frFj0=OqFfea0dXdd9vqai=hGuQ8kuc9pgc9s8qqaq=dirpe0xb9q8qiLsFr0=vr0=vr0dc8meaabaqaciaacaGaaeqabaqabeGadaaakeaacqWGtbWudaWgaaWcbaGaem4Ba8gabeaakiabg2da9maalaaabaWaaSaaaeaacqWGnbqtdaWgaaWcbaGaem4Ba8gabeaaaOqaaiabd6eaonaaBaaaleaacqWGVbWBaeqaaaaaaOqaamaalaaabaGaemyta00aaSbaaSqaaiabd+gaVbqabaaakeaacqWGobGtdaWgaaWcbaGaem4Ba8gabeaaaaGccqGHRaWkdaWcaaqaaiabd2eannaaBaaaleaacqWGKbazaeqaaaGcbaGaemOta40aaSbaaSqaaiabdsgaKbqabaaaaaaakiabbccaGiabbggaHjabb6gaUjabbsgaKjabbccaGiabdofatnaaBaaaleaacqWGKbazaeqaaOGaeyypa0JaeGymaeJaeyOeI0Iaem4uam1aaSbaaSqaaiabd+gaVbqabaGccqGGSaalcaWLjaGaaCzcamaabmaabaGaeGymaedacaGLOaGaayzkaaaaaa@5485@

where *M*_*o *_and *M*_*d *_are the occurrences of a given pattern in the ordered and disordered regions, and *N*_*o *_and *N*_*d *_are the total numbers of *k*-residue-long segments in the ordered regions and disordered regions. It is clearly that both *S*_*o *_and *S*_*d *_fall within 0 and 1, and if *S*_*o *_= *S*_*d *_= 0.5, the pattern has no preference for order or disorder. On the other hand, patterns with *S*_*o *_= 0 and *S*_*d *_= 1 and patterns with *S*_*o *_= 1 and *S*_*d *_= 0 are only observed in disordered and ordered regions respectively, and thus are considered as the most differential sequence patterns.

In this paper, only property patterns with three elements are considered since the occurrences of longer patterns might not be frequent enough to have statistical meanings. Table 7 lists the occurrences and scores for each pattern that is composed of three identical elements of a property group. Four clusters of property groups that are demonstrated to be useful in protein order/disorder prediction are considered here. In Table 7, the occurrences of the synthetic His purification tags present in both training and testing data have been excluded because those successive Hs bias the statistics of the observed patterns in this experiment, especially in the case of property group *Aromatic*. The results reveal that both the patterns composed of *Hydrophobic*_*O *_and *Hydrophobic*_*D *_are more differential than the pattern of three successive *Hydrophobic *residues. For the property groups *Polar*, *Small *and *Aromatic*, it is observed that at least one of the two child properties in each property group deliver the patterns that are more discriminative than the pattern of the parent property. This reveals the benefit of decomposing the conventional physicochemical properties when detecting differential patterns.

## Conclusion

More and more disordered regions are discovered in protein sequences, and many of them are found to be functionally significant. Distinguishing disordered regions from ordered regions in protein sequences facilitates the exploration of protein structures and functions. The evolutionary information embedded in the PSSM tables has been demonstrated useful in many problems that predict the functional or structural properties of a given residue in protein sequences. Our results in this paper demonstrate that considering the condensed PSSM with physicochemical properties and large window sizes further benefits the performance of protein disorder prediction.

The second and the most remarkable contribution of this paper is it removes some amino acid members from the widely used physicochemical properties that are not useful in predicting the disordered regions. This is achieved by decomposing each original physicochemical property into two disjoint sets that are with a propensity for order and disorder, respectively. In addition, a hybrid wrapped feature selection method that employs a uni-variant analysis and a cluster-based redundancy removal procedure is proposed to derive a satisfied feature set efficiently. Results on the independent testing data reveal that the proposed predicting model DisPSSMP outperforms the existing packages performing similar tasks without either under-predicting or over-predicting the disordered regions. Furthermore, the selected properties can be used to derive more informative patterns that facilitate the study of protein disorder. As more and more disordered regions are found to be functionally significant, combining predicted information of secondary structures and conversed regions for predicting disordered regions with binding ability deserves further studies.

## Methods

In this section, we provide the details about the procedures of constructing PSSMPs, calculating the propensities for order/disorder of an amino acid, training a predicting model, and selecting useful feature sets respectively.

### Construction of PSSMP

**Figure 8:**
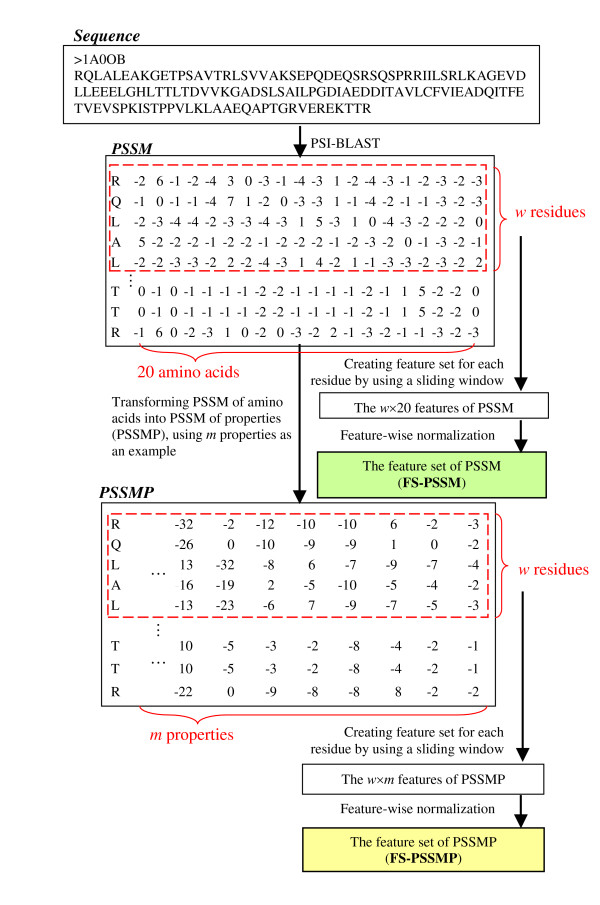
The procedure of preparing feature set for training and testing data.

For each protein in the training and testing data, we employ the PSI-BLAST program [43] to construct its position specific scoring matrix (PSSM). More specifically, the derived PSSM table is a position specific scoring matrix of 20 amino acids, which provides the evolutionary information about the protein at the level of residue types. We name the feature set created based on PSSMs FS-PSSM, which is considered as the baseline of employing evolutionary information in protein disorder prediction. The procedure of constructing FS-PSSM is shown in Figure 8. The values in PSSMs, which each represents the likelihood of a particular residue substitution at specific position, are first rescaled to be within 0 and 1 using the logistic function as suggested in [44]:

f(x)=11+exp⁡(−x),     (2)
 MathType@MTEF@5@5@+=feaafiart1ev1aaatCvAUfKttLearuWrP9MDH5MBPbIqV92AaeXatLxBI9gBaebbnrfifHhDYfgasaacH8akY=wiFfYdH8Gipec8Eeeu0xXdbba9frFj0=OqFfea0dXdd9vqai=hGuQ8kuc9pgc9s8qqaq=dirpe0xb9q8qiLsFr0=vr0=vr0dc8meaabaqaciaacaGaaeqabaqabeGadaaakeaacqWGMbGzcqGGOaakcqWG4baEcqGGPaqkcqGH9aqpdaWcaaqaaiabigdaXaqaaiabigdaXiabgUcaRiGbcwgaLjabcIha4jabcchaWjabcIcaOiabgkHiTiabdIha4jabcMcaPaaacqGGSaalcaWLjaGaaCzcamaabmaabaGaeGOmaidacaGLOaGaayzkaaaaaa@41EF@

where *x *is the raw value in profile matrices and *f*(*x*) is the rescaled value of *x*. After that, the rescaled profiles are organized into a number of *w *× 20 dimensional vectors, each of which serves as the feature vector of a target residue as the learning or predicting instances. When *w *is odd, which is always the case in our experiments, the sliding window of size *w *for acquiring the feature set of a given residue is centred at the target residue.

We next construct the feature set of PSSMP as follows. First, columns in the original PSSMs are grouped by the user defined property groups and the raw values from different columns are summed up as a new feature column. In a PSSMP table, the entry *y*_*ik *_of position *i *for property *k *is defined as follows:

yik=∑j=120Akj×xij,     (3)
 MathType@MTEF@5@5@+=feaafiart1ev1aaatCvAUfKttLearuWrP9MDH5MBPbIqV92AaeXatLxBI9gBaebbnrfifHhDYfgasaacH8akY=wiFfYdH8Gipec8Eeeu0xXdbba9frFj0=OqFfea0dXdd9vqai=hGuQ8kuc9pgc9s8qqaq=dirpe0xb9q8qiLsFr0=vr0=vr0dc8meaabaqaciaacaGaaeqabaqabeGadaaakeaacqWG5bqEdaWgaaWcbaGaemyAaKMaem4AaSgabeaakiabg2da9maaqahabaGaemyqae0aaSbaaSqaaiabdUgaRjabdQgaQbqabaaabaGaemOAaOMaeyypa0JaeGymaedabaGaeGOmaiJaeGimaadaniabggHiLdGccqGHxdaTcqWG4baEdaWgaaWcbaGaemyAaKMaemOAaOgabeaakiabcYcaSiaaxMaacaWLjaWaaeWaaeaacqaIZaWmaiaawIcacaGLPaaaaaa@48A3@

where

(1) *i *is the index of a position;

(2) *A*_*kj *_= 1, if the *j*-th type of amino acid belong in the *k*-th property, and *A*_*kj *_= 0, otherwise;

(3) *x*_*ij *_is the raw value of the *j*-th type of amino acid in the position *i *of the PSSM.

The concept of building PSSMP is also exemplified in Figure 8, and we call the derived feature set as FS-PSSMP. As will be explained the following subsection, different FS-PSSMPs can be generated when different property groups are specified in constructing FS-PSSMP.

### Considering propensity for order or disorder

Table 8 lists the ten physicochemical groups that are widely used in analyzing protein sequences [5,45]. This paper proposes considering the propensity for order or disorder of each amino acid when designing a property group in construction of PSSMPs. The propensities for order/disorder of different amino acids have been widely discussed in the previous studies [13,17,19,21,22,39-42]. Some of them specifically provide a measure of propensity based on the occurrences of each amino acid in different regions of the datasets they collected [22,39,41]. In this work, we recalculate the propensity for each amino acid based our training data. The propensity *P*(*AA*_*i*_) of an amino acid *i *toward ordered or disordered regions is defined as follows:

P(AAi)=FO(AAi)−FD(AAi)F(AAi),     (4)
 MathType@MTEF@5@5@+=feaafiart1ev1aaatCvAUfKttLearuWrP9MDH5MBPbIqV92AaeXatLxBI9gBaebbnrfifHhDYfgasaacH8akY=wiFfYdH8Gipec8Eeeu0xXdbba9frFj0=OqFfea0dXdd9vqai=hGuQ8kuc9pgc9s8qqaq=dirpe0xb9q8qiLsFr0=vr0=vr0dc8meaabaqaciaacaGaaeqabaqabeGadaaakeaacqWGqbaucqGGOaakcqWGbbqqcqWGbbqqdaWgaaWcbaGaemyAaKgabeaakiabcMcaPiabg2da9maalaaabaGaemOray0aaSbaaSqaaiabd+eapbqabaGccqGGOaakcqWGbbqqcqWGbbqqdaWgaaWcbaGaemyAaKgabeaakiabcMcaPiabgkHiTiabdAeagnaaBaaaleaacqWGebaraeqaaOGaeiikaGIaemyqaeKaemyqae0aaSbaaSqaaiabdMgaPbqabaGccqGGPaqkaeaacqWGgbGrcqGGOaakcqWGbbqqcqWGbbqqdaWgaaWcbaGaemyAaKgabeaakiabcMcaPaaacqGGSaalcaWLjaGaaCzcamaabmaabaGaeGinaqdacaGLOaGaayzkaaaaaa@4FC2@

where *F*(*AA*_*i*_) is the frequency of amino acid *i *in the training data and *F*_*O*_(*AA*_*i*_) and *F*_*D*_(*AA*_*i*_) are the frequencies of amino acid *i *in the ordered and disordered regions of the training data. We say amino acid *i *has a propensity for order if *P*(*AA*_*i*_) > 0, and verse visa.

**Figure 9:**
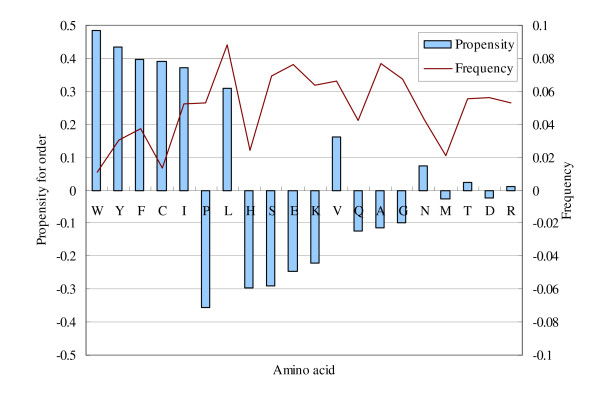
The propensity for order and the frequency of each amino acid in the training set.

We provide in Figure 9 the frequency of each amino acid in the training data, and the propensity for order *P*(*AA*_*i*_). The records shown in Figure 9 are compared with several previous studies [6,22,39,41]. For most amino acids, the propensity adopted in this paper is consistent with the preference of at least one of the previous studies. However, for the amino acids H, A, and M, the propensity for order/disorder is not certain according to the experimental results and the information collected at hand. More attention has been paid on the amino acid H, since some synthetic His tags present in the training data somewhat bias the statistics provided in Figure 9. Even though, we still adopt that H has a propensity for disorder because of its charged characteristic.

Based on the records shown in Figure 9, each physicochemical property in Table 8 can be decomposed into two disjoint set as new order/disorder-based property features, as shown in Table 9. Three exceptions are: *Aliphatic *has only three types of amino acid toward ordered regions, and *Negative *and *Proline *have only the disorder type of amino acids. It is noticed that there are some new properties which only comprise a single type of amino acid, such as *Aromatic*_*D*_, *Positive*_*O*_, *Charged*_*O*_, and *Tiny*_*O*_. All the property features will be considered in constructing the PSSMP feature set for protein disorder prediction. Since the size of the feature set is quite large and we do not expect all the property features are useful in predicting protein disorder, a feature selection method will be conducted to find a combination of property features that benefits protein disorder prediction.

### Classifier

In this study, the Radial Basis Function Network (RBFN) is used as the classifier for predicting protein disorder. The employed QuickRBF package [46] is an efficient tool for constructing RBFN classifiers, which uses the Cholesky decomposition technique to resolve the least mean square error optimization problem when constructing a RBFN classifier. We rely on the efficiency of QuickRBF such that a wrapped method of feature selection can be used in constructing our predictor DisPSSMP, where the 'wrapped' means that the classifier is employed in feature selection process for evaluating the predicting power of the candidate feature set [47].

According to the statistics provided in Table 1, the ratios of disorder residues to order residues in datasets PDB693 and D184 are 1:3.83 and 1:2.03, respectively. In order to tackle the problem of the skewed datasets with unbalanced number of positive and negative instances, equal quantity of residues from ordered and disordered regions is used in constructing the classifier. In other words, the same amount of ordered residues as that of the disordered residues in the training sets is randomly selected and the others are removed before the training process.

### Feature selection

It is doubted that all of the properties described in Table 8 and Table 9 are useful in the problem of disorder prediction. Thus, it is suggested to conduct a procedure of feature selection on the training data to find a combination of features that perform the best in this problem. Feature selection is a common optimization problem for finding the smallest subset of the features with the best classification performance [48]. However, finding the optimal feature subset is not easy, since there are 2^*n *^possible combinations when given *n *features. The algorithm of evaluating all subsets such as exhaustive search is impractical for large *n*. Therefore, an alternative stepwise feature selection is presented in this paper that takes the characteristics of features into account to improve the computational efficiency. Three factors are frequently used in evaluating the performance of a feature selection approach: classification accuracy, size of the subset, and computational efficiency [48]. The proposed hybrid method employs the efficient uni-variant analysis first, and uses a cluster-based redundancy removal procedure to speed up the tedious stepwise feature selection that explores the predicting power of combinations of multiple features simultaneously.

**Figure 10:**
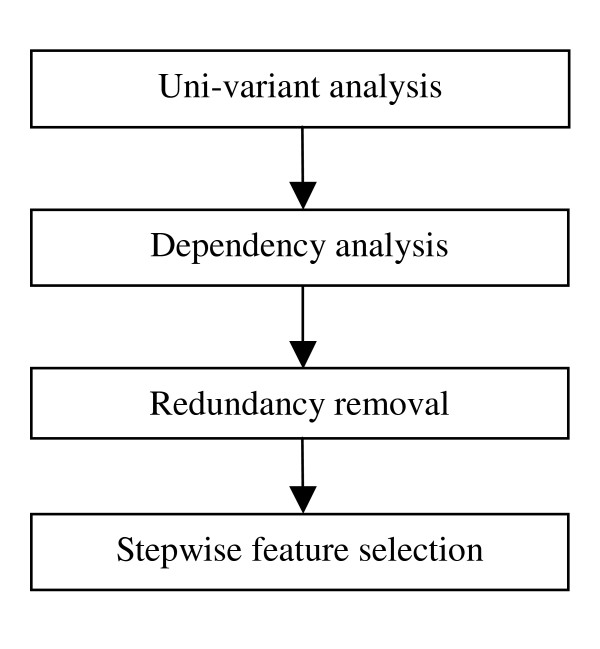
The flowchart of the hybrid feature selection mechanism.

Figure 10 shows the four steps of the proposed feature selection mechanism. First, uni-variant analyses are performed by conducting cross-validation on the training data using PSSMPs with one property at a time. After that the dependency analysis is executed in two levels. Since the members of properties listed in Table 8 and Table 9 are clearly specified, it is easy to put the related features in one cluster. The first level considers the dependency between the child properties in Table 9 and their parent properties in Table 8, and the second level considers the hierarchy dependency between the physicochemical properties listed in Table 8. After the dependency analysis, the redundancy removal step brings one feature or the combination of two features that performs the best in each cluster to the next step, stepwise feature selection. The representative properties from each cluster are sorted by their performance, and the final subset is constructed by adding property features one by one until the performance of cross validation on the training data cannot be improved.

## Availability and requirements

The executable file:  or 

Operating system(s): Linux

Programming language: C/Perl

Dependant packages: BLAST

Licence: None

Any restrictions to use by non-academics: None

## Abbreviations

PSSM, position specific scoring matrix; PSSMP, condensed position specific scoring matrix with respect to physicochemical properties; PDB, Protein Data Bank; NN, neural networks; LR, logistic regression; DA, discriminant analysis; CASP, the critical assessment of techniques for protein structure prediction; TP, true positive; FP, false positive; TN, true negative; FN, false negative; RBFN, radial basis function networks.

## Authors' contributions

CTS initialized the study and performed all calculations and analysis, and drafted the manuscript. CYC aided in design of the methodology, interpretation of the data, and manuscript preparation. YYO participated in the integration of the RBFN classifier. All authors read and approved the manuscript.

## Supplementary Material

Additional File 1 This supplement provides the complete version of Table 2, 3, 4, 5, 6 , the records involved in Figure 5, 6, 7, and the protein list of the training data. Click here for file

## Figures and Tables

**Table 1:** Summary of the datasets employed in this study

	Training data		Testing data		
	
	PDB693	D184	R80	U79	P80
Number of chains	693	184	80	79	80
Number of ordered regions	1357	257	151	0	80
Number of disordered regions	1739	274	183	79	0
Number of residues in the ordered regions	201937	55164	29909	0	16568
Number of residues in the disordered regions	52663	27116	3649	14462	0
Total residues in the dataset	254600	82280	33558	14462	16568

**Table 2:** The definition of measures employed in this study

Measure	Abbreviation	Equation *
Sensitivity (recall)	*Sens*.	TP/(TP+FN)
Specificity	*Spec*.	TN/(TN+FP)
Matthews' correlation coefficient	*MCC*	(TP×TN-FP×FN)/sqrt((TP+FP)×(TN+FN)×(TP+FN)×(TN+FP))
Probability excess	*Prob. Excess*	(TP×TN-FP×FN)/((TP+FN)×(TN+FP))

* The definition of the abbreviations used: TP is the number of correctly classified disordered residues; FP is the number of ordered residues incorrectly classified as disordered; TN is the number of correctly classified ordered residues; and FN is the number of disordered residues incorrectly classified as ordered.

**Table 3:** The performance of each property in the uni-variant analysis on training data

Property	*Sens*.	*Spec*.	*MCC*	*Prob. Excess*
*Hydrophobic*	0.633	0.717	0.309	0.350
***Hydrophobic***_*O*_	**0.640**	**0.751**	**0.350**	**0.391**
*Hydrophobic*_*D*_	0.519	0.723	0.217	0.241

***Polar***	**0.616**	**0.734**	**0.312**	**0.350**
*Polar*_*O*_	0.603	0.703	0.269	0.306
*Polar*_*D*_	0.604	0.731	0.299	0.335

*Small*	0.553	0.742	0.268	0.295
*Small*_*O*_	0.555	0.688	0.214	0.243
***Small***_*D*_	**0.579**	**0.759**	**0.308**	**0.338**

***Aliphatic***	**0.601**	**0.748**	**0.314**	**0.349**

*Aromatic*	0.604	0.720	0.288	0.324
***Aromatic***_*O*_	**0.602**	**0.732**	**0.298**	**0.334**
*Aromatic*_*D*_	0.538	0.660	0.173	0.198

***Positive***	**0.599**	**0.678**	**0.242**	**0.277**
*Positive*_*O*_	0.573	0.662	0.204	0.235
*Positive*_*D*_	0.583	0.667	0.218	0.250

***Negative***	**0.586**	**0.696**	**0.248**	**0.282**

***Proline***	**0.564**	**0.684**	**0.218**	**0.248**

***Charged***	**0.614**	**0.707**	**0.282**	**0.320**
*Charged*_*O*_	0.571	0.664	0.204	0.235
*Charged*_*D*_	0.603	0.706	0.272	0.309

*Tiny*	0.528	0.732	0.234	0.259
*Tiny*_*O*_	0.577	0.675	0.220	0.252
***Tiny***_*D*_	**0.553**	**0.748**	**0.274**	**0.301**

The best performance among each property group is highlighted with bold font.

**Table 4:** Performance evaluation on *Hydrophobic*, *Aliphatic*, and *Aromatic*

Property	*Sens*.	*Spec*.	*MCC*	*Prob. Excess*
*Hydrophobic*_*O*_	0.640	0.751	0.350	0.391
*Aliphatic*	0.601	0.748	0.314	0.349
*Aromatic*_*O*_	0.602	0.732	0.298	0.334
***Aliphatic + Aromatic***_*O*_	**0.645**	**0.768**	**0.373**	**0.413**

The best performance is highlighted with bold font.

**Table 5:** Performance evaluation on *Polar*, *Charged*, *Positive*, and *Negative*

Property	*Sens*.	*Spec*.	*MCC*	*Prob. Excess*
***Polar***	**0.616**	**0.734**	**0.312**	**0.350**
*Charged*	0.614	0.707	0.282	0.320
*Positive*	0.599	0.678	0.242	0.277
*Negative*	0.586	0.696	0.248	0.282
*Positive + Negative*	0.607	0.715	0.284	0.321

The best performance is highlighted with bold font.

**Table 6:** The result of the stepwise feature selection

Property	*Sens*.	*Spec*.	*MCC*	*Prob. Excess*
*Aliphatic*+*Aromatic*_*O*_	0.646	0.767	0.372	0.412
*Aliphatic*+*Aromatic*_*O*_+*Polar*	0.656	0.774	0.390	0.430
***Aliphatic*+*Aromatic***_*O*_**+*Polar*+*Small***_*D*_	**0.656**	**0.781**	**0.397**	**0.437**
*Aliphatic*+*Aromatic*_*O*_+*Polar*+*Small*_*D*_+*Proline*	0.652	0.783	0.396	0.435

The best performance is highlighted with bold font.

**Table 7:** The statistics of the property patterns with three identical residues in the ordered and disordered regions

Patterns	# of matches in # subsequences				Score	
			
	In ordered regions		In disordered regions			
	
	# matches	# seqs	# matches	# seqs	*S*_*o*_	*S*_*d*_
[*Hydrophobic*] [*Hydrophobic*] [*Hydrophobic*]	71229	2087	16534	1555	**0.56**	0.44
[*Hydrophobic*_*O*_] [*Hydrophobic*_*O*_] [*Hydrophobic*_*O*_]	14608	1746	2297	687	**0.65**	0.35
[*Hydrophobic*_*D*_] [*Hydrophobic*_*D*_] [*Hydrophobic*_*D*_]	4766	1224	2649	789	0.35	**0.65**

[*Polar*] [*Polar*] [*Polar*]	38084	1965	16390	1585	0.41	**0.59**
[*Polar*_*O*_] [*Polar*_*O*_] [*Polar*_*O*_]	2530	927	762	372	**0.50**	0.50
[*Polar*_*D*_] [*Polar*_*D*_] [*Polar*_*D*_]	8808	1459	5705	1060	0.31	**0.69**

[*Small*] [*Small*] [*Small*]	37886	1949	15577	1630	0.42	**0.58**
[*Small*_*O*_] [*Small*_*O*_] [*Small*_*O*_]	2050	824	569	284	**0.52**	0.48
[*Small*_*D*_] [*Small*_*D*_] [*Small*_*D*_]	9742	1504	5662	1169	0.34	**0.66**

[*Aromatic*] [*Aromatic*] [*Aromatic*]	467	302	54	47	**0.72**	0.28
[*Aromatic*_*O*_] [*Aromatic*_*O*_] [*Aromatic*_*O*_]	219	159	20	17	**0.76**	0.24
[*Aromatic*_*D*_] [*Aromatic*_*D*_] [*Aromatic*_*D*_]	5	5	4	4	0.27	**0.73**

Improvements in discriminating power are highlighted with bold font.

**Table 8:** Conventional Amino Acid Properties (Parent Properties)

Property	I	L	V	C	A	G	M	F	Y	W	H	K	R	E	Q	D	N	S	T	P
*Hydrophobic*	Y	Y	Y	Y	Y	Y	Y	Y	Y	Y	Y	Y							Y	
*Polar*									Y	Y	Y	Y	Y	Y	Y	Y	Y	Y	Y	
*Small*			Y	Y	Y	Y										Y	Y	Y	Y	Y
*Aliphatic*	Y	Y	Y																	
*Aromatic*								Y	Y	Y	Y									
*Positive*											Y	Y	Y							
*Negative*														Y		Y				
*Proline*																				Y
*Charged*											Y	Y	Y	Y		Y				
*Tiny*				Y	Y	Y												Y		

**Table 9:** Order/Disorder-based Amino Acid Properties (Child Properties)

Property	I	L	V	C	A	G	M	F	Y	W	H	K	R	E	Q	D	N	S	T	P
*Hydrophobic*_*O*_	Y	Y	Y	Y				Y	Y	Y									Y	
*Hydrophobic*_*D*_					Y	Y	Y				Y	Y								
*Polar*_*O*_									Y	Y			Y				Y		Y	
*Polar*_*D*_											Y	Y		Y	Y	Y		Y		
*Small*_*O*_			Y	Y													Y		Y	
*Small*_*D*_					Y	Y										Y		Y		Y
*Aliphatic*_O_*	Y	Y	Y																	
*Aromatic*_*O*_								Y	Y	Y										
*Aromatic*_*D *_^#^											Y									
*Positive*_*O *_^#^													Y							
*Positive*_*D*_											Y	Y								
*Negative*_D_*														Y		Y				
*Proline*_D_*^#^																				Y
*Charged*_*O *_^#^													Y							
*Charged*_*D*_											Y	Y		Y		Y				
*Tiny*_*O *_^#^				Y																
*Tiny*_*D*_					Y	Y												Y		

* *Aliphatic*_*O*_, *Negative*_*D*_, and *Proline*_*D *_are equivalent to *Aliphatic*, *Negative*, and *Proline *in Table 8, respectively.

# *Aromatic*_*D*_, *Positive*_*O*_, *Proline*_*D*_, *Charged*_*O*_, and *Tiny*_*O *_each comprises only a single type of amino acid.
