# Supplementary material for: Protein disorder prediction by condensed PSSM considering propensity for order or disorder
Source: BMC Bioinformatics. 2006 Jun 23;7:319. doi: 10.1186/1471-2105-7-319 (PMC1526762; doi:10.1186/1471-2105-7-319)
Supplement: Additional File 1 — This supplement provides the complete version of Table 2, 3, 4, 5, 6 , the records involved in Figure 5, 6, 7, and the protein list of the training data. [file 1471-2105-7-319-S1.doc]

# Protein disorder prediction by condensed PSSM considering propensity for order or disorder

# Supplement

Chung-Tsai Su^1^, Chien-Yu Chen[[1]](#footnote-2)*2, and Yu-Yen Ou^3,4^

^1^ Department of Computer Science and Information Engineering, National Taiwan University, Taipei, 106, Taiwan, R.O.C.
^2^ Department of Bio-industrial Mechatronics Engineering, National Taiwan University, Taipei, 106, Taiwan, R.O.C.
^3^ Graduate School of Biotechnology and Bioinformatics, Yuan Ze University, Chung-Li, 320, Taiwan, R.O.C.
^4^ Department of Computer Science and Engineering, Yuan Ze University, Chung-Li, 320, Taiwan, R.O.C.

sbb@mars.csie.ntu.edu.tw, cychen@mars.csie.ntu.edu.tw, yien@csie.org

This supplement provides the complete version of Table 2-6 and the records involved in Figure 5-7 of the manuscript, as well as the protein lists in the training data. The contents are segmented into four sections.

More evaluation measures

Table Suppl.I provides the definition of the symbols used in Table Suppl.II that lists eight evaluation measures adopted in this study. *Sensitivity* represents the fraction of disordered residues correctly identified in a prediction, while *specificity* indicates the fraction of ordered residues correctly identified. For integrating *sensitivity* and *specificity*, *accuracy* represents the fraction of disordered and ordered residues correctly identified in a prediction. In addition, the *Matthews’ correlation coefficient* is a popular measure in many bioinformatics problems. However, *accuracy* and the *Matthews’ correlation coefficient* are seriously affected by the relative class frequency. The *probability excess*, *CASP S score*, and *product* are recommended and advised by Yang *et al*. and CASP6. Since these three measures have the same tendency with *probability excess*, we only adopt the *probability excess* for evaluating predicting packages in the manuscript, and provide the complete results here.

Results on feature selection

Uni-variant analysis is executed as the first step of the feature selection. The complete results are sorted by the measure of *probability excess* and shown in Table Suppl.III. In the same way, the complete results regarding the dependency analysis and stepwise feature selection are shown in Table Suppl.IV, , and Table Suppl.VI.

Results on testing data

In comparison with other methods, DisPSSMP performs the best in the first blind testing dataset when the three measures *CASP S score*, *produce*, and *probability excess* are concerned, as shown in Table Suppl.VII. Meanwhile, DisPSSMP gets the second rank on the second blind testing dataset as shown in Table Suppl.VIII. The overall results on the blind testing data are organized in Table Suppl.IX.

Protein lists of training datasets

At the end of this document, we provide the entry name of each protein in the training datasets PDB693 and D184 in Table Suppl.X and Table Suppl.XI, respectively.

1. The symbols used in Table Suppl.II

| Name | Acronym | Description |
| --- | --- | --- |
| True positive | TP | The number of correctly classified disordered residues |
| False positive | FP | The number of ordered residues incorrectly classified as disordered |
| True negative | TN | The number of correctly classified ordered residues |
| False negative | FN | The number of disordered residues incorrectly classified as ordered |
| Weight of TP | wTP | The number of ordered residues divided by the total number of residues |
| Weight of FP | wFP | - _wTN_ |
| Weight of TN | wTN | The number of disordered residues divided by the total number of residues |
| Weight of FN | wFN | - _wTP_ |

1. The equations of evaluation measures (the complete version of Table 2)

| Measure | Abbreviation | Equality |
| --- | --- | --- |
| Sensitivity (Recall) | *Sens.* | TP/(TP+FN) |
| Specificity | *Spec.* | TN/(TN+FP) |
| Precision | *Prec.* | TP/(TP+FP) |
| Accuracy | *Accu.* | (TP+TN)/(TP+FP+TN+FN) |
| Matthews' correlation coefficient | *MCC* | (TP×TN-FP×FN)/sqrt((TP+FP)×(TN+FN)×(TP+FN)×(TN+FP)) |
| CAPS S Score | *CASP S* | (_wTP_×TP+wFP×FP+wTN×TN+wFN×FN)/( _wTP_× (TP +FN)+ wTN× (TN +FP)) |
| Product | *Prod.* | (TP×TN)/((TP+FN)×(TN+FP)) |
| Probability excess | *Prob. Excess* | (TP×TN-FP×FN)/((TP+FN)×(TN+FP)) |

1. The performance of each amino acid property by uni-variant analysis (the complete version of Table 3)

| Property | TP | FP | TN | FN | *Sens.* | *Spec.* | *Prec.* | *Accu.* | *MCC* | *CASP S* | *Prod.* | *Prob. Excess* |
| --- | --- | --- | --- | --- | --- | --- | --- | --- | --- | --- | --- | --- |
| *Hydrophobic* | 50506 | 72749 | 184352 | 29273 | 0.633 | 0.717 | 0.410 | 0.697 | 0.309 | 12.66 | 0.454 | 0.350 |
| ***Hydrophobic_O_*** | **51081** | **64086** | **193015** | **28698** | **0.640** | **0.751** | **0.444** | **0.725** | **0.350** | **14.13** | **0.481** | **0.391** |
| *Hydrophobic_D_* | 41370 | 71288 | 185813 | 38409 | 0.519 | 0.723 | 0.367 | 0.674 | 0.217 | 8.72 | 0.375 | 0.241 |
| ***Polar*** | **49124** | **68400** | **188701** | **30655** | **0.616** | **0.734** | **0.418** | **0.706** | **0.312** | **12.64** | **0.452** | **0.350** |
| *Polar_O_* | 48126 | 76463 | 180638 | 31653 | 0.603 | 0.703 | 0.386 | 0.679 | 0.269 | 11.06 | 0.424 | 0.306 |
| *Polar_D_* | 48178 | 69036 | 188065 | 31601 | 0.604 | 0.731 | 0.411 | 0.701 | 0.299 | 12.12 | 0.442 | 0.335 |
| *Small* | 44130 | 66278 | 190823 | 35649 | 0.553 | 0.742 | 0.400 | 0.697 | 0.268 | 10.68 | 0.411 | 0.295 |
| *Small_O_* | 44295 | 80194 | 176907 | 35484 | 0.555 | 0.688 | 0.356 | 0.657 | 0.214 | 8.79 | 0.382 | 0.243 |
| ***Small_D_*** | **46215** | **62019** | **195082** | **33564** | **0.579** | **0.759** | **0.427** | **0.716** | **0.308** | **12.22** | **0.440** | **0.338** |
| *Aliphatic* | 47909 | 64785 | 192316 | 31870 | 0.601 | 0.748 | 0.425 | 0.713 | 0.314 | 12.60 | 0.449 | **0.349** |
| *Aromatic* | 48224 | 71983 | 185118 | 31555 | 0.604 | 0.720 | 0.401 | 0.693 | 0.288 | 11.73 | 0.435 | 0.324 |
| ***Aromatic_O_*** | **48010** | **68911** | **188190** | **31769** | **0.602** | **0.732** | **0.411** | **0.701** | **0.298** | **12.06** | **0.440** | **0.334** |
| *Aromatic_D_* | 42893 | 87398 | 169703 | 36886 | 0.538 | 0.660 | 0.329 | 0.631 | 0.173 | 7.15 | 0.355 | 0.198 |
| ***Positive*** | **47750** | **82703** | **174398** | **32029** | **0.599** | **0.678** | **0.366** | **0.659** | **0.242** | **10.01** | **0.406** | **0.277** |
| *Positive_O_* | 45686 | 86823 | 170278 | 34093 | 0.573 | 0.662 | 0.345 | 0.641 | 0.204 | 8.49 | 0.379 | 0.235 |
| *Positive_D_* | 46546 | 85724 | 171377 | 33233 | 0.583 | 0.667 | 0.352 | 0.647 | 0.218 | 9.04 | 0.389 | 0.250 |
| ***Negative*** | **46754** | **78102** | **178999** | **33025** | **0.586** | **0.696** | **0.374** | **0.670** | **0.248** | **10.20** | **0.408** | **0.282** |
| ***Proline*** | **44964** | **81124** | **175977** | **34815** | **0.564** | **0.684** | **0.357** | **0.656** | **0.218** | **8.97** | **0.386** | **0.248** |
| ***Charged*** | **48966** | **75459** | **181642** | **30813** | **0.614** | **0.707** | **0.394** | **0.685** | **0.282** | **11.58** | **0.434** | **0.320** |
| *Charged_O_* | 45547 | 86450 | 170651 | 34232 | 0.571 | 0.664 | 0.345 | 0.642 | 0.204 | 8.48 | 0.379 | 0.235 |
| *Charged_D_* | 48096 | 75644 | 181457 | 31683 | 0.603 | 0.706 | 0.389 | 0.681 | 0.272 | 11.16 | 0.425 | 0.309 |
| *Tiny* | 42103 | 69031 | 188070 | 37676 | 0.528 | 0.732 | 0.379 | 0.683 | 0.234 | 9.37 | 0.386 | 0.259 |
| *Tiny_O_* | 46014 | 83549 | 173552 | 33765 | 0.577 | 0.675 | 0.355 | 0.652 | 0.220 | 9.10 | 0.389 | 0.252 |
| ***Tiny_D_*** | **44087** | **64694** | **192407** | **35692** | **0.553** | **0.748** | **0.405** | **0.702** | **0.274** | **10.88** | **0.414** | **0.301** |

The best performance among each property group is highlighted with bold font.

1. The comparison between properties *Hydrophobic*, *Aliphatic*, and *Aromatic* (the complete version of Table 4)

| Property | TP | FP | TN | FN | *Sens.* | *Spec.* | *Prec.* | *Accu.* | *MCC* | *CASP S* | *Prod.* | *Prob. Excess* |
| --- | --- | --- | --- | --- | --- | --- | --- | --- | --- | --- | --- | --- |
| *Hydrophobic_O_* | 51081 | 64086 | 193015 | 28698 | 0.640 | 0.751 | 0.444 | 0.725 | 0.350 | 14.13 | 0.481 | 0.391 |
| *Aliphatic* | 47909 | 64785 | 192316 | 31870 | 0.601 | 0.748 | 0.425 | 0.713 | 0.314 | 12.60 | 0.449 | 0.349 |
| *Aromatic_O_* | 48010 | 68911 | 188190 | 31769 | 0.602 | 0.732 | 0.411 | 0.701 | 0.298 | 12.06 | 0.440 | 0.334 |
| ***Aliphatic + Aromatic_O_*** | **51463** | **59752** | **197349** | **28316** | **0.645** | **0.768** | **0.463** | **0.739** | **0.373** | **14.92** | **0.495** | **0.413** |

**The best performance is highlighted with bold font.**

1. The comparison between *Polar*, *Positive*, and *Negative* (the complete version of Table 5)

| Property | TP | FP | TN | FN | *Sens.* | *Spec.* | *Prec.* | *Accu.* | *MCC* | *CASP S* | *Prod.* | *Prob. Excess* |
| --- | --- | --- | --- | --- | --- | --- | --- | --- | --- | --- | --- | --- |
| ***Polar*** | **49124** | **68400** | **188701** | **30655** | **0.616** | **0.734** | **0.418** | **0.706** | **0.312** | **12.64** | **0.452** | **0.350** |
| *Positive* | 47750 | 82703 | 174398 | 32029 | 0.599 | 0.678 | 0.366 | 0.659 | 0.242 | 10.01 | 0.406 | 0.277 |
| *Negative* | 46754 | 78102 | 178999 | 33025 | 0.586 | 0.696 | 0.374 | 0.670 | 0.248 | 10.20 | 0.408 | 0.282 |
| *Positive + Negative* | 48386 | 73372 | 183729 | 31393 | 0.607 | 0.715 | 0.397 | 0.689 | 0.284 | 11.61 | 0.433 | 0.321 |

The best performance is highlighted with bold font.

1. Results of the stepwise feature selection (the complete version of Table 6)

| Property | TP | FP | TN | FN | *Sens.* | *Spec.* | *Prec.* | *Accu.* | *MCC* | *CASP S* | *Prod.* | *Prob. Excess* |
| --- | --- | --- | --- | --- | --- | --- | --- | --- | --- | --- | --- | --- |
| *Aliphatic+Aromatic_O_* | 51507 | 59998 | 197103 | 28272 | 0.646 | 0.767 | 0.462 | 0.738 | 0.372 | 14.90 | 0.495 | 0.412 |
| *Aliphatic+Aromatic_O_+Polar* | 52353 | 58076 | 199025 | 27426 | 0.656 | 0.774 | 0.474 | 0.746 | 0.390 | 15.55 | 0.508 | 0.430 |
| ***Aliphatic+Aromatic_O_+Polar+Small_D_*** | **52328** | **56309** | **200792** | **27451** | **0.656** | **0.781** | **0.482** | **0.751** | **0.397** | **15.79** | **0.512** | **0.437** |
| *Aliphatic+Aromatic_O_+Polar+Small_D_+Proline* | 52036 | 55847 | 201254 | 27743 | 0.652 | 0.783 | 0.482 | 0.752 | 0.396 | 15.72 | 0.511 | 0.435 |

The best performance is highlighted with bold font.

1. Comparing the performance of thirteen packages predicting protein disorder on the testing dataset R80. (the complete results of Figure 5)

| Method | TP | FP | TN | FN | *Sens.* | *Spec.* | *Prec.* | *Accu.* | *MCC* | *CASP S* | *Prod.* | *Prob. Excess* |
| --- | --- | --- | --- | --- | --- | --- | --- | --- | --- | --- | --- | --- |
| DisPSSMP | 2800 | 4550 | 25359 | 849 | 0.767 | 0.848 | 0.381 | 0.839 | 0.463 | 0.119 | 0.651 | 0.615 |
| RONN | 2200 | 3634 | 26275 | 1449 | 0.603 | 0.878 | 0.377 | 0.849 | 0.395 | 0.093 | 0.530 | 0.481 |
| IUPred(short) | 1887 | 1642 | 28267 | 1762 | 0.517 | 0.945 | 0.535 | 0.899 | 0.469 | 0.090 | 0.489 | 0.462 |
| DISpro | 1525 | 209 | 29700 | 2124 | 0.418 | 0.993 | 0.879 | 0.930 | 0.578 | 0.080 | 0.415 | 0.411 |
| IUPred(long) | 1591 | 1179 | 28730 | 2058 | 0.436 | 0.961 | 0.574 | 0.904 | 0.449 | 0.077 | 0.419 | 0.397 |
| DISOPRED2* | 1432 | 734 | 25890 | 2104 | 0.405 | 0.972 | 0.661 | 0.906 | 0.470 | 0.078 | 0.394 | 0.377 |
| PONDR | 2033 | 5518 | 24391 | 1616 | 0.557 | 0.816 | 0.269 | 0.787 | 0.278 | 0.072 | 0.454 | 0.373 |
| DisEMBL(hot) | 1795 | 4788 | 25121 | 1854 | 0.492 | 0.840 | 0.273 | 0.802 | 0.260 | 0.064 | 0.413 | 0.332 |
| DisEMBL(465) | 1217 | 564 | 29345 | 2432 | 0.334 | 0.981 | 0.683 | 0.911 | 0.437 | 0.061 | 0.327 | 0.315 |
| FoldIndex | 1782 | 5664 | 24245 | 1867 | 0.488 | 0.811 | 0.239 | 0.776 | 0.224 | 0.058 | 0.396 | 0.299 |
| PreLink | 863 | 1597 | 28312 | 2786 | 0.237 | 0.947 | 0.351 | 0.869 | 0.219 | 0.035 | 0.224 | 0.183 |
| GlobPlot | 1357 | 5654 | 24255 | 2292 | 0.372 | 0.811 | 0.194 | 0.763 | 0.140 | 0.035 | 0.302 | 0.183 |
| DisEMBL(coils) | 2702 | 17222 | 12687 | 947 | 0.740 | 0.424 | 0.136 | 0.459 | 0.104 | 0.032 | 0.314 | 0.165 |

*For DISOPRED2, the public web server has a sequence length limit of 1000 residues; therefore, 1HN0, 1FO4, and 1PS3 in R80 cannot be predicted.

1. Comparing the performance of thirteen packages predicting protein disorder on the testing datasets U79 and P80. (the complete results of Figure 6)

| Method | TP | FP | TN | FN | *Sens.* | *Spec.* | *Prec.* | *Accu.* | *MCC* | *CASP S* | *Prod.* | *Prob. Excess* |
| --- | --- | --- | --- | --- | --- | --- | --- | --- | --- | --- | --- | --- |
| IUPred(long) | 9807 | 963 | 15605 | 4655 | 0.678 | 0.942 | 0.911 | 0.819 | 0.650 | 0.309 | 0.639 | 0.620 |
| DisPSSMP | 11934 | 3896 | 12672 | 2528 | 0.825 | 0.765 | 0.754 | 0.793 | 0.589 | 0.294 | 0.631 | 0.590 |
| RONN | 9763 | 1854 | 14714 | 4699 | 0.675 | 0.888 | 0.840 | 0.789 | 0.580 | 0.280 | 0.600 | 0.563 |
| FoldIndex | 10439 | 3071 | 13497 | 4023 | 0.722 | 0.815 | 0.773 | 0.771 | 0.540 | 0.267 | 0.588 | 0.536 |
| IUPred(short) | 8047 | 1406 | 15162 | 6415 | 0.556 | 0.915 | 0.851 | 0.748 | 0.511 | 0.235 | 0.509 | 0.472 |
| DISPRED2* | 5921 | 318 | 16250 | 6714 | 0.469 | 0.981 | 0.949 | 0.759 | 0.543 | 0.221 | 0.460 | 0.449 |
| PONDR | 9139 | 3611 | 12957 | 5323 | 0.632 | 0.782 | 0.717 | 0.712 | 0.420 | 0.206 | 0.494 | 0.414 |
| DISpro | 5540 | 292 | 16276 | 8922 | 0.383 | 0.982 | 0.950 | 0.703 | 0.467 | 0.182 | 0.376 | 0.365 |
| DisEMBL(465) | 5039 | 359 | 16209 | 9423 | 0.348 | 0.978 | 0.933 | 0.685 | 0.430 | 0.163 | 0.341 | 0.327 |
| PreLink | 4608 | 141 | 16427 | 9854 | 0.319 | 0.991 | 0.970 | 0.678 | 0.430 | 0.154 | 0.316 | 0.310 |
| DisEMBL(hot) | 7263 | 4159 | 12409 | 7199 | 0.502 | 0.749 | 0.636 | 0.634 | 0.260 | 0.125 | 0.376 | 0.251 |
| DisEMBL(coils) | 10398 | 9182 | 7386 | 4064 | 0.719 | 0.446 | 0.531 | 0.573 | 0.170 | 0.082 | 0.321 | 0.165 |
| GlobPlot | 4461 | 2970 | 13598 | 10001 | 0.308 | 0.821 | 0.600 | 0.582 | 0.151 | 0.064 | 0.253 | 0.129 |

*For DISOPRED2, the public web server has a sequence length limit of 1000 residues; therefore, the u15 protein in U79 cannot be predicted.

1. Comparing the performance of thirteen packages predicting protein disorder on the testing datasets R80, U79, and P80. (the complete results of Figure 7)

| Method | TP | FP | TN | FN | *Sens.* | *Spec.* | *Prec.* | *Accu.* | *MCC* | *CASP S* | *Prod.* | *Prob. Excess* |
| --- | --- | --- | --- | --- | --- | --- | --- | --- | --- | --- | --- | --- |
| **DisPSSMP** | **14734** | **8446** | **38031** | **3377** | **0.814** | **0.818** | **0.636** | **0.817** | **0.592** | **0.255** | **0.666** | **0.632** |
| IUPred(long) | 11398 | 2142 | 44335 | 6713 | 0.629 | 0.954 | 0.842 | 0.863 | 0.644 | 0.235 | 0.600 | 0.583 |
| RONN | 11963 | 5488 | 40989 | 6148 | 0.661 | 0.882 | 0.686 | 0.820 | 0.549 | 0.219 | 0.583 | 0.542 |
| FoldIndex | 12221 | 8735 | 37742 | 5890 | 0.675 | 0.812 | 0.583 | 0.774 | 0.467 | 0.196 | 0.548 | 0.487 |
| IUPred(short) | 9934 | 3048 | 43429 | 8177 | 0.549 | 0.934 | 0.765 | 0.826 | 0.541 | 0.195 | 0.513 | 0.483 |
| DISOPRED2* | 7353 | 1052 | 42140 | 8818 | 0.455 | 0.976 | 0.875 | 0.834 | 0.550 | 0.171 | 0.444 | 0.430 |
| PONDR | 11172 | 9129 | 37348 | 6939 | 0.617 | 0.804 | 0.550 | 0.751 | 0.407 | 0.170 | 0.496 | 0.420 |
| DISpro | 7065 | 501 | 45976 | 11046 | 0.390 | 0.989 | 0.934 | 0.821 | 0.530 | 0.153 | 0.386 | 0.379 |
| DisEMBL(465) | 6256 | 923 | 45554 | 11855 | 0.345 | 0.980 | 0.871 | 0.802 | 0.465 | 0.131 | 0.339 | 0.326 |
| DisEMBL(hot) | 9058 | 8947 | 37530 | 9053 | 0.500 | 0.807 | 0.503 | 0.721 | 0.308 | 0.124 | 0.404 | 0.308 |
| PreLink | 5471 | 1738 | 44739 | 12640 | 0.302 | 0.963 | 0.759 | 0.777 | 0.378 | 0.107 | 0.291 | 0.265 |
| DisEMBL(coils) | 13100 | 26404 | 20073 | 5011 | 0.723 | 0.432 | 0.332 | 0.514 | 0.143 | 0.063 | 0.312 | 0.155 |
| GlobPlot | 5818 | 8624 | 37853 | 12293 | 0.321 | 0.814 | 0.403 | 0.676 | 0.146 | 0.055 | 0.262 | 0.136 |

*For DISOPRED2, the public web server has a sequence length limit of 1000 residues; therefore, 1HN0, 1FO4, and 1PS3 in R80 and the u15 protein in U79 cannot be predicted.

1. The list of the proteins in the training dataset PDB693

| PDB693 | | | | | | |
| --- | --- | --- | --- | --- | --- | --- |
| 16VPA | 1FFTB | 1JFIB | 1NG0A | 1QQ0A | 1TMO | 1XLXA |
| 1A0OB | 1FGJA | 1JHFB | 1NHZA | 1QQGA | 1TOAA | 1XMAA |
| 1A22B | 1FHGA | 1JIIA | 1NJ1A | 1QS1A | 1TOLA | 1XMJA |
| 1A36A | 1FIWA | 1JJ2G | 1NKTA | 1QVHH | 1TQQA | 1XMRA |
| 1A37A | 1FJGC | 1JK0A | 1NKWE | 1QWYA | 1TT0A | 1XOUA |
| 1A5LC | 1FJMA | 1JK0B | 1NKWF | 1QX7D | 1TTWB | 1XQEA |
| 1A81E | 1FKAD | 1JKFA | 1NKWN | 1QY6A | 1TVLA | 1XRFA |
| 1AGQA | 1FKAR | 1JKGB | 1NLZC | 1QYUA | 1TWYA | 1XRJA |
| 1AGRE | 1FKMA | 1JM6A | 1NMBN | 1QZ2A | 1TXNA | 1XRSB |
| 1AL0B | 1FNTU | 1JMAB | 1NO1A | 1QZ7B | 1TYQC | 1XTEA |
| 1AL3 | 1FQYA | 1JMAA | 1NOVA | 1R0VA | 1U04A | 1XVIA |
| 1AMUA | 1FS9A | 1JMJA | 1NRGA | 1R27B | 1U0RB | 1XVLA |
| 1AMX | 1FSTA | 1JMUB | 1NRIA | 1R30A | 1U2JA | 1XWSA |
| 1AO7D | 1FUUA | 1JNK | 1NT9D | 1R52A | 1U4FA | 1XZQA |
| 1AROP | 1FXZA | 1JPHA | 1NW1A | 1R5MA | 1U67A | 1Y08A |
| 1ATIA | 1G0UM | 1JQOA | 1NW3A | 1R6TB | 1U6GC | 1Y10A |
| 1AUIA | 1G3JC | 1JQPA | 1NYHA | 1R71A | 1U78A | 1Y1OA |
| 1B34A | 1G5GA | 1JSQA | 1NZEA | 1R7RA | 1U7FB | 1Y44A |
| 1B3OA | 1G5RA | 1JSWC | 1O4ZA | 1RDR | 1U9IC | 1Y4SA |
| 1B70A | 1G6IA | 1JV2B | 1O5DL | 1REWC | 1U9OA | 1Y6AA |
| 1B89A | 1G8EB | 1JXQA | 1O5DT | 1RGQA | 1UA2A | 1Y80A |
| 1B8MB | 1G9RA | 1K3VA | 1O5LA | 1RI1A | 1UD0D | 1Y8QB |
| 1B9XC | 1G9UA | 1K3ZD | 1O6OD | 1RIID | 1UE1A | 1YA0A |
| 1BB9 | 1GCYA | 1K5DB | 1O91A | 1RJGA | 1UF2A | 1YAEF |
| 1BCCB | 1GD2J | 1K5DC | 1O94D | 1RJKA | 1UF2K | 1YBXA |
| 1BE3I | 1GIYE | 1K6IA | 1OATA | 1RJMA | 1UIJC | 1YBYA |
| 1BG1A | 1GJVA | 1K78I | 1OD2A | 1RK8A | 1UL1Y | 1YC1A |
| 1BGW | 1GK9A | 1K87A | 1OD5A | 1RLUA | 1UN0C | 1YCSB |
| 1BGYE | 1GMEB | 1K8KB | 1OE9A | 1RO8B | 1UNGD | 1YD7A |
| 1BI2B | 1GMNB | 1KCXA | 1OEDA | 1RQEA | 1URSB | 1YDHB |
| 1BIF | 1GRH | 1KGNA | 1OEDB | 1RQGA | 1US7B | 1YEWC |
| 1BIIA | 1GV4A | 1KKTA | 1OEDC | 1RV2A | 1UTBA | 1YGUA |

**Table Suppl.X. (Continued)** The list of the proteins in the training dataset PDB693

| PDB693 | | | | | | |
| --- | --- | --- | --- | --- | --- | --- |
| 1BMFG | 1GW5A | 1KMIZ | 1OEDE | 1RXTA | 1V02A | 1YISA |
| 1BMP | 1GZ0E | 1KMMA | 1OF5A | 1RY6A | 1V0DA | 1YJ5C |
| 1BO1A | 1GZHD | 1KMOA | 1OF5B | 1RY7B | 1V5WA | 1YM7A |
| 1C5KA | 1H0NA | 1KO6A | 1OFTA | 1RYFA | 1V8DA | 1YMMD |
| 1C8BA | 1H2AS | 1KO6B | 1OHFA | 1RZ2A | 1V8JA | 1YMYB |
| 1C8DA | 1H2DA | 1KOHB | 1OHHH | 1RZNA | 1V98A | 1YNUA |
| 1C8M4 | 1H2VZ | 1KOQA | 1OHTA | 1S1EA | 1V9DC | 1YOXD |
| 1C8NA | 1H3NA | 1KPLA | 1OIUC | 1S1ID | 1V9YA | 1YQ7A |
| 1CBF | 1H4TB | 1KT0A | 1OJLB | 1S1IN | 1VCRA | 1YR2A |
| 1CD1A | 1H6KB | 1KTKF | 1OLZA | 1S1IW | 1VDDC | 1YTFC |
| 1CHUA | 1H6WA | 1KWPA | 1ONFA | 1S1IX | 1VDZA | 1YTVM |
| 1CJKC | 1H76A | 1KXF | 1OPLA | 1S1IY | 1VE5C | 1YTZI |
| 1CJMA | 1H7UA | 1KY9A | 1OPOA | 1S21A | 1VF7A | 1YU6C |
| 1CJYA | 1H89C | 1KZQA | 1ORHA | 1S2JA | 1VFGA | 1YVGA |
| 1CLC | 1H8EH | 1L0OC | 1OT3A | 1S3RA | 1VH3A | 1YWHA |
| 1CLWA | 1HBXG | 1L3AA | 1OT8B | 1S3SI | 1VH6A | 1Z05A |
| 1CO7I | 1HCNB | 1L5HA | 1OU5A | 1S4EG | 1VHKD | 1Z4VA |
| 1CP3A | 1HFES | 1L8AA | 1OVLA | 1S5JA | 1VK5A | 1Z5VA |
| 1CT9A | 1HK7A | 1L8KA | 1OW3A | 1S5LC | 1VKYA | 1Z5XE |
| 1CWPA | 1HNKA | 1L8WA | 1OXNA | 1S72I | 1VL5C | 1Z6KA |
| 1D2HA | 1HU3A | 1L9BM | 1P4DA | 1S72Y | 1VLRA | 1Z6UA |
| 1D2MA | 1HVUG | 1L9ZH | 1P5JA | 1S78A | 1VP3 | 1Z7DC |
| 1D2QA | 1HVXA | 1LAJA | 1P5SA | 1S80A | 1VPGA | 1Z81A |
| 1D9XA | 1HW4A | 1LARB | 1P6FA | 1S94A | 1VQUB | 1Z92B |
| 1DAR | 1HYNP | 1LAY | 1P6GF | 1S9HC | 1VR9A | 1Z9FA |
| 1DD7A | 1HYQA | 1LBD | 1P6GN | 1S9IB | 1VRDA | 1ZCUA |
| 1DEQA | 1HZFA | 1LBHA | 1P7BA | 1SCFA | 1VRWA | 1ZE2B |
| 1DF0A | 1HZTA | 1LD4M | 1P85B | 1SE8A | 1VYHC | 1ZIWA |
| 1DGSA | 1I0IA | 1LI5A | 1P85C | 1SERA | 1VYVA | 1ZNNA |
| 1DIOB | 1I2MA | 1LOX | 1P85F | 1SEVA | 1VZOA | 1ZTMA |
| 1DIOG | 1I3QA | 1LSHA | 1P85J | 1SG2B | 1W1OA | 1ZTPC |
| 1DKGA | 1I3QC | 1LSHB | 1P85O | 1SGJA | 1W1WA | 1ZXEB |
| 1DKIA | 1I3QF | 1LTLA | 1P8CA | 1SHKB | 1W1WE | 1ZY9A |

**Table Suppl.X. (Continued)** The list of the proteins in the training dataset PDB693

| PDB693 | | | | | | |
| --- | --- | --- | --- | --- | --- | --- |
| 1DLYA | 1I41A | 1LTXR | 1P99A | 1SHSA | 1W2B5 | 2A11A |
| 1DMGA | 1I4OC | 1LUFA | 1P9EA | 1SJNA | 1W36D | 2A1TA |
| 1DP5B | 1I7DA | 1LW3A | 1PF4A | 1SJPA | 1W36F | 2A33B |
| 1DPI | 1I7FA | 1M0FF | 1PJAA | 1SMVA | 1W46A | 2A3LA |
| 1DVEA | 1I84S | 1M0UA | 1PJR | 1SO2A | 1W6UC | 2A3QA |
| 1DZLA | 1I8NA | 1M1HA | 1PK8A | 1SP9A | 1W7PD | 2A79B |
| 1E1HA | 1I94M | 1M2VB | 1PKYC | 1SQ1A | 1W81A | 2BBVA |
| 1E3HA | 1IBJA | 1M41A | 1PME | 1SQBA | 1W8XP | 2BHVA |
| 1E5RA | 1ID3D | 1M6BA | 1POV1 | 1SR4A | 1W9PA | 2BIVA |
| 1E94E | 1IIPA | 1M9SA | 1PQ4A | 1SR9A | 1WAWA | 2BJUA |
| 1E9NA | 1IK6A | 1MB1 | 1PQVS | 1SRQD | 1WB1B | 2BKIA |
| 1EEPA | 1IK7A | 1MCXA | 1PSCA | 1SVCP | 1WDJB | 2BNXA |
| 1EFM | 1IKOP | 1MEYG | 1PSDB | 1SW6A | 1WK2A | 2BOVB |
| 1EG2A | 1IR6A | 1MG7A | 1PU4A | 1SXJA | 1WM9A | 2BP1A |
| 1EH7A | 1IS7A | 1MH5B | 1PVOB | 1SY6A | 1WNCA | 2BTOA |
| 1EI3A | 1IVOA | 1MHPY | 1PWPB | 1SZIA | 1WP1B | 2BTVA |
| 1EI3B | 1IW7A | 1ML5E | 1PZDA | 1SZWA | 1WV4A | 2CAUA |
| 1EJ6B | 1IW7D | 1MNNA | 1PZNA | 1T10A | 1X6VB | 2CSMA |
| 1EQQA | 1IXRA | 1MQ8A | 1PZSA | 1T3EP | 1X7UA | 2GNKA |
| 1EW2A | 1IYJB | 1MQBA | 1Q0CA | 1T3JA | 1X9DA | 2IG2H |
| 1EWRA | 1IZLA | 1MQLB | 1Q14A | 1T4HB | 1X9NA | 2PF1 |
| 1EYSC | 1IZLB | 1MQSA | 1Q1CA | 1T4OA | 1X9PA | 2PRGC |
| 1EZXC | 1IZLD | 1MU7A | 1Q1LA | 1T5AA | 1XA6A | 2TBVA |
| 1F0XA | 1IZLE | 1MVFD | 1Q1SC | 1T6PB | 1XARA | 2TS1 |
| 1F15A | 1J3IA | 1MVMA | 1Q32A | 1T7LA | 1XDIA | 4HB1 |
| 1F1JA | 1J9YA | 1N0HA | 1Q3DA | 1T94B | 1XDTR | 4SBVA |
| 1F1OA | 1JA0B | 1N0YA | 1Q55A | 1T9GR | 1XFVA | 5CRXB |
| 1F2NA | 1JB7A | 1N3LA | 1Q67A | 1TBGB | 1XHOC | 7CEIB |
| 1F66A | 1JB7B | 1N4KA | 1Q8IA | 1TDHA | 1XI8A |  |
| 1F66D | 1JBQA | 1N6DA | 1QAXB | 1TEDB | 1XIOA |  |
| 1F7CA | 1JC9A | 1N7DA | 1QB3A | 1TF2A | 1XJ5A |  |
| 1F8VB | 1JCHA | 1N93X | 1QBZB | 1TG6A | 1XJDA |  |
| 1FCBB | 1JCQA | 1N9EA | 1QE0B | 1TH1C | 1XK8A |  |

**Table Suppl.X. (Continued)** The list of the proteins in the training dataset PDB693

| PDB693 | | | | | | |
| --- | --- | --- | --- | --- | --- | --- |
| 1FCHA | 1JDPA | 1NAEA | 1QHUA | 1TLLB | 1XKKA |  |
| 1FFTA | 1JEQA | 1NFUB | 1QMEA | 1TME4 | 1XKSA |  |

1. The list of the proteins in the training dataset D184

| D184 | | | | | | |
| --- | --- | --- | --- | --- | --- | --- |
| DP00001 | DP00031 | DP00065 | DP00098 | DP00142 | DP00175 | DP00217 |
| DP00002 | DP00032 | DP00066 | DP00100 | DP00143 | DP00177 | DP00218 |
| DP00003 | DP00033 | DP00067 | DP00102 | DP00144 | DP00179 | DP00219 |
| DP00004 | DP00034 | DP00068 | DP00103 | DP00145 | DP00180 | DP00221 |
| DP00005 | DP00036 | DP00069 | DP00108 | DP00146 | DP00181 | DP00222 |
| DP00006 | DP00038 | DP00070 | DP00109 | DP00147 | DP00182 | DP00223 |
| DP00007 | DP00039 | DP00071 | DP00110 | DP00148 | DP00184 | DP00224 |
| DP00008 | DP00040 | DP00072 | DP00112 | DP00149 | DP00190 | DP00225 |
| DP00010 | DP00041 | DP00075 | DP00113 | DP00150 | DP00191 | DP00227 |
| DP00011 | DP00042 | DP00076 | DP00116 | DP00151 | DP00192 | DP00228 |
| DP00012 | DP00044 | DP00077 | DP00117 | DP00152 | DP00193 | DP00229 |
| DP00013 | DP00045 | DP00078 | DP00118 | DP00154 | DP00197 | DP00230 |
| DP00014 | DP00046 | DP00080 | DP00119 | DP00155 | DP00198 | DP00232 |
| DP00015 | DP00048 | DP00082 | DP00120 | DP00156 | DP00199 | DP00233 |
| DP00016 | DP00049 | DP00083 | DP00121 | DP00157 | DP00201 | DP00234 |
| DP00017 | DP00050 | DP00084 | DP00124 | DP00158 | DP00203 | DP00235 |
| DP00018 | DP00052 | DP00085 | DP00125 | DP00159 | DP00205 | DP00236 |
| DP00019 | DP00053 | DP00087 | DP00126 | DP00160 | DP00206 | DP00238 |
| DP00021 | DP00054 | DP00088 | DP00127 | DP00161 | DP00207 | DP00239 |
| DP00022 | DP00056 | DP00089 | DP00128 | DP00162 | DP00208 | DP00240 |
| DP00024 | DP00057 | DP00090 | DP00130 | DP00163 | DP00209 | DP00241 |
| DP00025 | DP00058 | DP00091 | DP00132 | DP00164 | DP00210 | DP00242 |
| DP00026 | DP00059 | DP00092 | DP00136 | DP00167 | DP00211 |  |
| DP00027 | DP00061 | DP00093 | DP00137 | DP00169 | DP00213 |  |
| DP00028 | DP00062 | DP00094 | DP00138 | DP00171 | DP00214 |  |
| DP00029 | DP00063 | DP00095 | DP00140 | DP00173 | DP00215 |  |

1. ^^ Corresponding author: Chien-Yu Chen, Eamil: cychen@mars.csie.ntu.edu.tw, Tel: +886-2-33665334, Fax: +886-2-23627620, Postal address: Dept. of Bio-industrial Mechatronics Engineering, National Taiwan University, No.1, Sec. 4, Roosevelt Rd., Taipei, 106, Taiwan (R.O.C.) [↑](#footnote-ref-2)
